# Supplementary material for: A Critical View of the Application of the APEX Software (Aqueous Photochemistry of Environmentally-Occurring Xenobiotics) to Predict Photoreaction Kinetics in Surface Freshwaters
Source: Molecules. 2019 Dec 18;25(1):9. doi: 10.3390/molecules25010009 (PMC7017383; doi:10.3390/molecules25010009)
Supplement: Supplementary file 1 [file molecules-25-00009-s001.zip › 3.2.4_gcc-4.4.0.zip!/3.2.4_gcc-4.4.0/3.2.4_gcc-4.4.0/doc/octave/html/interpreter/Actual-Bugs.html]

Actual Bugs - Untitled


Next: Reporting Bugs,
Up: Trouble


---

### E.1 Actual Bugs We Haven't Fixed Yet

- Output that comes directly from Fortran functions is not sent through
  the pager and may appear out of sequence with other output that is sent
  through the pager. One way to avoid this is to force pending output to
  be flushed before calling a function that will produce output from
  within Fortran functions. To do this, use the command

  ```
            fflush (stdout)
  ```

  Another possible workaround is to use the command

  ```
            page_screen_output (false);
  ```

  to turn the pager off.

A list of ideas for future enhancements is distributed with Octave. See
the file PROJECTS in the top level directory in the source
distribution.
